# New HIV diagnoses in patients with COVID-19: two case reports and a brief literature review

**DOI:** 10.1186/s12879-020-05480-y

**Published:** 2020-10-19

**Authors:** Jiu-Cong Zhang, Xiao-Hui Yu, Xiao-Han Ding, Hao-Yu Ma, Xiao-Qing Cai, Sheng-Chao Kang, Da-Wei Xiang

**Affiliations:** 1 The 940 Hospital of Joint Logistic Support Force of PLA, Lanzhou, 730050 China; 2 Huoshenshan Hospital, Wuhan, 430050 China

**Keywords:** SARS-CoV-2, COVID-19, Co-infection, HIV, Case report, Literature review

## Abstract

**Background:**

COVID-19 is novel infectious disease with an evolving understanding of its epidemiology and clinical manifestations. Severe cases developed life-threatening complications, such as respiratory failure, shock, and multiple organs dysfunction. Immunocompromised patients often present atypical presentations of viral infected diseases.

**Case presentation:**

We report newly diagnosed HIV infections in two patients with COVID-19 in China. In our two cases, both patients with elevated IL-6 received Tocilizumab treatment, but did not present obvious therapeutic effect.

**Conclusions:**

These cases highlight possible co-detection of known immunocompromised diseases such as HIV. The two cases we reported stressed the risk of misdiagnosis, especially during the pandemic of an infectious disease and the importance of extended testing even if in immune-compromised condition the immune state may be ignored.

## Background

An outbreak of pneumonia of unknown origin was first reported in Wuhan, China in December 2019. The cause had been identified as severe acute respiratory syndrome coronavirus 2 (SARS-CoV-2), which was officially named as COVID-19 (coronavirus disease 2019) by World Health Organization (WHO), could induce symptoms including fever, dry cough, dyspnea, fatigue, and lymphopenia and ground-glass lung changes in radiology in infected patients. The pandemic of COVID-19 has posed great threat to public health across the globe. Until April 21, 2020, 2,397,217 cases were confirmed globally, including 84,250 cases in China [1]. A total of 162,956 patients have died from this viral infection [2]. Severe cases developed life-threatening complications, such as respiratory failure, shock, and multiple organs dysfunction [3]. We report co-infection of severe acute respiratory syndrome coronavirus 2 (SARS-CoV-2) and HIV in two patients in China.

## Case presentation

### Case one

**Fig. 1:**
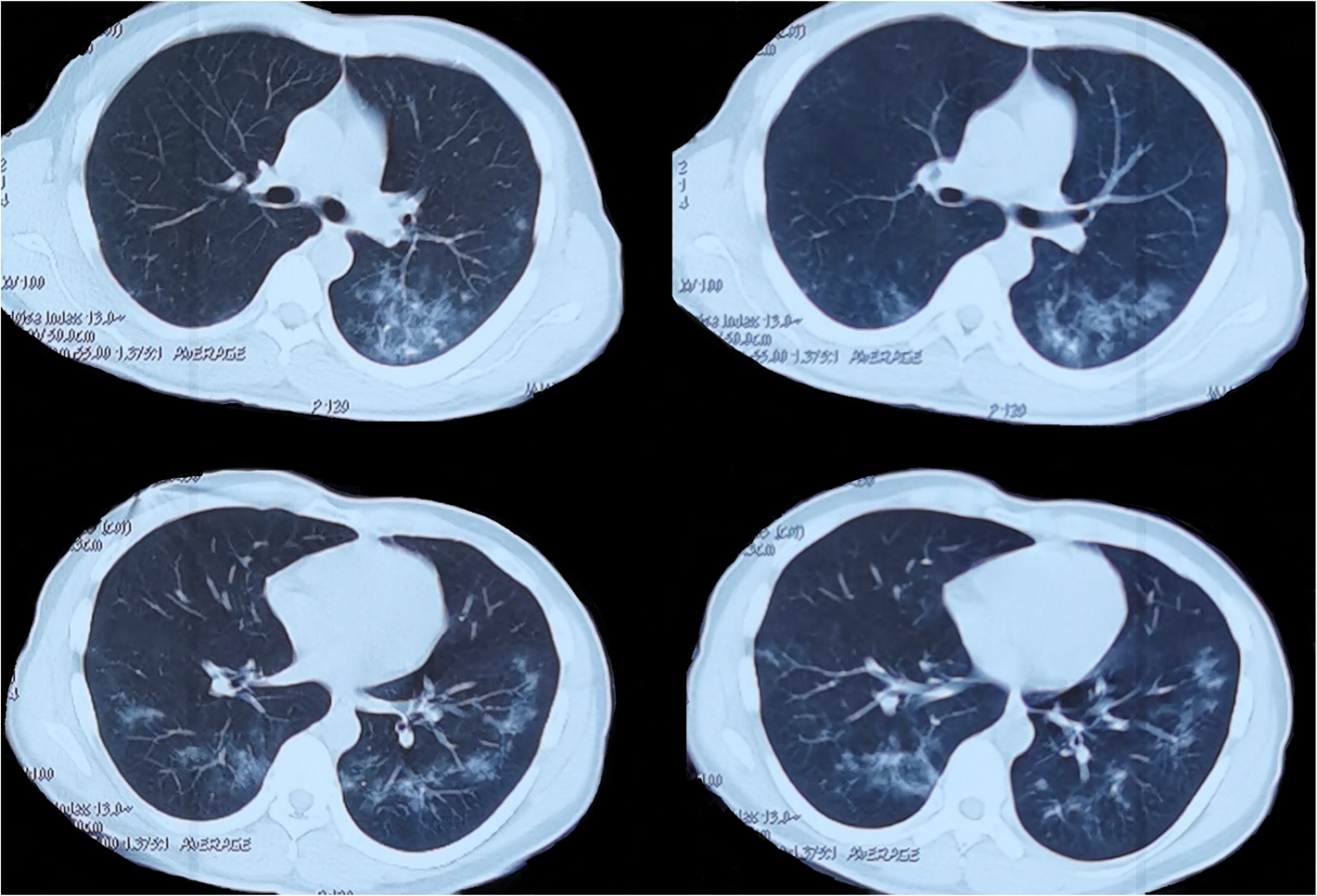
Chest CT images of patient one on Feb 9

A 24-year-old man was seen in the clinic of a local Hospital on February 8, 2020, for fever with a maximum body temperature of 40 °C, accompanied by fatigue, poor appetite, dizziness. In the past half month, the body weight decreased by 2.5 kg. The patient lived in Wuhan and began having symptoms on February 8. A COVID-19 was diagnosed by SARS-CoV-2 RT-PCR came back positive on February 8 and a chest CT examination, which suggested interstitial lung disease on February 9 (Fig. 1). He was then hospitalized, but his symptoms of fever, chest tightness and shortness of breath were not significantly improved after the symptomatic treatment. On February 18, chest computed tomography revealed ground-glass opacities, which were mainly on the periphery of the lungs (supplementary Fig. 2). Then the patient was transferred to our hospital on February 20 (Table 1). He reported no underlying medical conditions. There was neither blood transfusion nor intravenous drug abuse (sharing of non-sterilized needles). However, it was likely that the patient was a man who has sex with men (MSM).

**Table 1:** Events and timeline of the disease course in patient one in co-infection of HIV and SARS-CoV-2

Hospitalization	Day1	Day2	Day3	Day4	Day5	Day6	Day7	Day8	Day9	Day10	Day11	Day12	Day13	Day14	Day15	Day16	Day17	Day18	Day19	Day20	Day21	Day22	Day23	Day24	Day25	Day26	Day27	Day28	Day29	Day49
Fever, °C	36.5	37	37	37	37	38.5	37.4	37.2	38	37	37.1	37.1	40	36.8	36.7	37	36.6	37	36.5	36.5	36.5	36.5	36.6	37	37.2	36.8	37.2	36.4	36.5	
Nasopharyngeal swabs											N										N	N								
WBC counts, ×10^9^/L		6.3						5.0						6.9				4.3						7.8						
Lymphocyte, × 10^9^/L		1.08						1.16						1.20				0.70						1.31						
Platelet, × 10^12^/L		285						273						272				249						210						
CRP, mg/L		39.71																						0.29						
Procalcitonin, ng/ml		0.03																0.01						0.02						
Albumin, g/L		38.2						36.2										33.1				32.7		32.5						
Interlukin-6, pg/mL													30.54							688.40					521.00					
SARS-CoV-2 IgG															63.52										3.87					
SARS-CoV-2 IgM															30.12										0.96					
CD4 T cells/μL																									13					
imaging findings					GGO							GGO,								GGO,					GGO,,some become solic					
HIV viral load (copies/mL)																														1.7 × 10^5^

Abbreviations: *COVID-19* Coronavirus disease 2019; *CRP* C-reactive protein; *GGO* Ground-glass opacities; *HIV* Human immunodeficiency virus; *IgM* Immunoglobulin M; *WBC* White blood cell; *N* Negative

Routine blood tests revealed a leukocyte count of 6.3 × 10^9^ cells/L (reference range 3.5–9.5 × 10^9^ cells/L) and lymphocyte count of 1.08 × 10^9^ cells/L (reference range 1.1–3.2 × 10^9^ cells/L), lymphocyte percentage 17.0% (reference range 20–50%), C-reactive protein 39.71 mg/L (reference range 0–4 mg/L), hypersensitive C-reactive protein > 10.00 mg/L (reference range 0–4 mg/L). Biochemical test: albumin 38.2 g/L (reference range 40–55 g/L).

The main clinical manifestations were intermittent low fever, night sweat and sore throat. Physical examination showed that the pharynx was congested and swollen, and the tonsil was swollen at grade II. After admission, the patient was given antiviral therapy with Arbidol.

On day 4 of hospitalization, chest computed tomography revealed increased ground-glass opacities (Supplementary file 1). There is no improvement compared with previous CT examination. The patient received moxifloxacin 400 mg once daily for 7 days then he felt relief of sore throat but continuous night sweat and intermittent low fever. SARS-CoV-2 RT-PCR was performed on his throat swabs on day 10, which confirmed a negative result.

On day 12, chest computed tomography revealed ground-glass opacities which were slightly larger than previous one (supplementary Fig. 3). At the night of day 13, the patient’s temperature rose to 40 °C, and then fell down after symptomatic treatment.

On day 13, interleukin 6 was 30.54 pg/mL (reference range < 7 pg/mL), then the patient was treated with Tocilizumab on day 14, together with immunoglobulin and thymosin. After that, there was no fever and night sweat present, but symptom of pharyngeal pain, chest tightness and shortness of breath increased. Physical examination was still found that the tonsil was swollen in degree II and the tonsil was ulcerated.

On day 15, serum SARS-CoV-2 antibody indicated IgM 30.12 (reference range < 10) and IgG 63.52 (reference range < 10). Chest computed tomography on day 20 revealed the texture of both lungs increased, and ground-glass opacities increased in both lungs (supplementary Fig. 4). On day 20, interleukin 6 examination was 688.40 pg/mL. The range of pulmonary lesions increased comparing with CT on day 12.

SARS-CoV-2 RT-PCR assay for detection of coronavirus RNA were performed on his throat swabs on day 21 and day 22 with negative results.

We calculated the percentage change trend of lymphocytes and monocytes and found that after a short period of transient increase, the percentage of lymphocytes showed a gradual and slow downward trend; the percentage of monocytes first increased slowly, and then also showed a slow downward trend (supplementary Fig. 5). The decreasing trend of albumin seemed plain (supplementary Fig. 6).

On day 23, an antigen/antibody combination test on blood gave a HIV-positive result and a *Treponema pallidum* positive result. The patient’s chest tightness and shortness of breath worsen on day 25 with a progressed CT lesion of both lungs, the density of some lesions became solid (supplementary Fig. 7). We compared the infection percentage of the three CT examinations in hospital by calculating the infection volume through the artificial intelligence technology of chest CT, and we found that the lung infection volume of the patients increased in an equal proportion (supplementary Fig. 8).

On day 25, interleukin 6 examination was 521.00 pg/mL, serum SARS-CoV-2 antibody indicated a reversal. A T cell subsets analysis indicated that the CD4^+^ T cell count is 13 cells/μL (reference range 500–1600 cells/μL). According to the inter department consultation, the patient’s pulmonary infection was not exclusive of *Pneumocystis jirovecii* Pneumonia, tuberculosis and cytomegalovirus infection, and then the patient was transfer to a designated hospital for further treatment on day 29.

### Case two

A 37-year-old man was referred to our hospital on February 11, 2020 due to fever for more than 1 month (Table 2). At the beginning of January 2020, the patient had fever, the maximum temperature is 39.5 °C, during which chest pain occurred intermittently. In February, chest CT from a local hospital showed multiple exudation in both lungs (Figure lost), and RT-PCR assay for the detection of SARS-CoV-2 was performed on a nasopharyngeal swab and returned negative. The main symptoms of the patient after admission were obvious wheezing after activity which can gradually improve after rest. After admission, the patient was given antiviral therapy with Arbidol.

**Table 2:** Events and timeline of the disease course in patient two in co-infection of HIV and SARS-CoV-2

Hospitalization	Day1	Day2	Day3	Day4	Day5	Day6	Day7	Day8	Day9	Day10	Day11	Day12	Day13	Day14	Day15	Day16	Day17	Day18	Day19	Day20	Day21	Day22	Day23	Day24	Day25	Day26	Day27	Day47
Fever, °C	38.8	38.1	38.7	39.4	37	38.6	36.6	36.9	36.8	36.7	36.8	38.1	36.7	36.7	36.6	36.7	36.4	37.5	36.7	37.5	37.1	37.4	37	36.8	37.2	37	37.5	
Nasopharyngeal swabs										N					N				N			N						
WBC counts, ×10^9^/L		4.2					3.2				4.6				3.8						3.3							
Lymphocyte, ×10^9^/L		1.55					0.60				0.91				0.84						0.56							
Platelet, ×10^12^/L		267					233				289				196						143							
CRP, mg/L		96.51					42.70				26.06				11.14						11.65							
Procalcitonin, ng/ml							0.04																					
Albumin, g/L		33.2									28.7				27.5						26							
Interlukin-6, pg/mL																						9.87				141.40		
SARS-CoV-2 IgG																									0.63			
SARS-CoV-2 IgM																									0.79			
imaging findings										GGO									GGO									
HIV viral load (copies/mL)																												9.7 × 10^4^

Abbreviations: *COVID-19* Coronavirus disease 2019; *CRP* C-reactive protein; *GGO* Ground-glass opacities; *HIV* Human immunodeficiency virus; *IgM* Immunoglobulin M; *WBC* White blood cell; *N*, negative

Routine blood tests revealed a leukocyte count of 4.2 × 10^9^ cells/L (reference range 3.5–9.5 × 10^9^ cells/L) and lymphocyte count of 1.55 × 10^9^ cells/L (reference range 1.1–3.2 × 10^9^ cells/L), lymphocyte percentage 36.8% (reference range 20–50%), C-reactive protein 96.51 mg/L (reference range 0–4 mg/L), hypersensitive C-reactive protein > 10.00 mg/L (reference range 0–4 mg/L). Biochemical test: albumin 33.2 g/L (reference range 40–55 g/L).

On day 4, the patient had fever at night. Tmax was 39.4 °C, and there was still obvious panting. When panting, the heart rate was fast, accompanied by palpitation. The patient also received moxifloxacin 400 mg once daily for 5 days, methilprednisolone 0.8 mg/kg once daily for 5 days through intravenous route.

On day 10, SARS-CoV-2 RT-PCR was performed on his throat swabs, which confirmed a negative result and chest computed tomography revealed there are multiple large, slightly high-density shadows in both lungs, mostly with ground glass like changes, mainly distributed in the middle and outer zones (supplementary Fig. 9). The patient showed a marked clinical and radiological improvement. His oxygen saturation measured by pulse maintained above 95% on supplemental oxygen via nasal cannula at 15 l per minute in resting state, but decreased rapidly after a little activity, the lowest was about 80%. The patient still felt chest pain, but no D-dimer elevation was found, and the bedside ECG examination also failed to indicate special changes. The cause of chest pain considered the possible involvement of pleura.

On day 11, routine blood tests revealed a leukocyte count of 4.6 × 10^9^ cells/L (reference range 3.5–9.5 × 10^9^ cells/L) and lymphocyte count of 0.91 × 10^9^ cells/L (reference range 1.1–3.2 × 10^9^ cells/L), lymphocyte percentage 19.8% (reference range 20–50%), C-reactive protein 26.06 mg/L (reference range 0–4 mg/L), hypersensitive C-reactive protein > 10.00 mg/L (reference range 0–4 mg/L). Biochemical test: albumin 28.7 g/L (reference range 40–55 g/L).

On day 15, routine blood tests revealed a leukocyte count of 3.8 × 10^9^ cells/L (reference range 3.5–9.5 × 10^9^ cells/L) and lymphocyte count of 0.84 × 10^9^ cells/L (reference range 1.1–3.2 × 10^9^ cells/L), lymphocyte percentage 22.1% (reference range 20–50%), C-reactive protein 11.14 mg/L (reference range 0–4 mg/L), hypersensitive C-reactive protein > 10.00 mg/L (reference range 0–4 mg/L). Biochemical test: albumin 27.5 g/L (reference range 40–55 g/L).

On day 19, chest computed tomography revealed the bilateral lung texture increased, the density of patchy and flocculent increased in large area, and some of them were ground glass like, which was better than that on day 10 (supplementary Fig. 10–11).

On day 21, routine blood tests revealed a leukocyte count of 3.3 × 10^9^ cells/L (reference range 3.5–9.5 × 10^9^ cells/L) and lymphocyte count of 0.56 × 10^9^ cells/L (reference range 1.1–3.2 × 10^9^ cells/L), lymphocyte percentage 17% (reference range 20–50%, supplementary Fig. 12), C-reactive protein 11.65 mg/L (reference range 0–4 mg/L, supplementary Fig. 13), hypersensitive C-reactive protein > 10.00 mg/L (reference range 0–4 mg/L). Biochemical test: albumin 26 g/L (reference range 40–55 g/L, supplementary Fig. 13).

Two times of SARS-CoV-2 RT-PCR assay for detection of coronavirus RNA were performed on his throat swabs on day 19 and day 22, which both confirmed negative results.

On day 22, the patient still has shortness of breath after obvious activity, and sputum is not easy to cough up. After the treatment of antiviral, the patient’s condition did not improve significantly, and there was no absorption of the disease in imaging, so it is possible to combine the infection of Gram-negative bacteria. However, due to the limited conditions, the etiological examination could not be improved, so cefoperazone sulbactam sodium was empirically given and acetylcysteine was given for expectorant treatment.

On day 24, the patient was treated with Tocilizumab together with administration of immunoglobulin and thymosin in that the effect of the previous treatment was not significantly improved and an assay for interleukin 6 was 9.87 pg/mL (day 22). After that, the patient still had intermittent fever, Tmax 37.5 °C. Under the resting state, the blood oxygen saturation was over 95% when given mask oxygen for 5 L/min, but the patient still felt obvious panting after activity.

On day 25, serum SARS-CoV-2 antibody indicated IgM 0.79 (reference range < 10) and IgG 0.63 (reference range < 10). On day 26, interleukin 6 examination was 141.40 pg/mL and an antigen/antibody combination test on blood gave a HIV-positive result and a *Treponema pallidum* positive result.

On day 27, the patient was asked to receive the antiretroviral treatment in a designated hospital, in light of local epidemic prevention law, until then a COVID-19 was confirmed by a positive SARS-CoV-2 IgM report and the T cell subsets analysis of the patient indicated that the CD4^+^ T cell count is 23 cells/μL.

## Discussion and conclusions

The first case of co-infection human immunodeficiency virus (HIV) and SARS-CoV-2 was reported in Wuhan, China, with the spread of the epidemic, subsequent cases have been reported in Italy, Turkey, Germany, USA, UK, Spain, Uganda, Japan and other countries [2].

HIV infection continues to be a major global health concern [4]. HIV and syphilis co-infection has been frequently observed worldwide [5]. It is well-established that the increase in cases of HIV has played an important role in the resurgence of syphilis, which, in turn, provides a favorable environment for HIV transmission [6]. However, in our cases we were unable to exclude the false-positive results occurred without rapid plasma reagin test (RPR) even an antibody positive test. Moreover, for the second patient we cannot exclude syphilis because he admitted that he once had a painless rash, and he did not answer questions about the history of sexual behavior.

There are rapidly growing information on COVID-19 infection, however, the data in HIV population remains contradictory [7, 8]. Previous studies observed that low CD4^+^ T cell counts could actually protect against severe form of COVID-19, which suggested that immune system activation may actually increase the injury caused by COVID-19 and low CD4^+^ T cell counts might protect HIV-positive individuals from developing the cytokine storm observed in patients with COVID-19 [9–13].

However, Suwanwongse et al observed the clinical features and outcomes of nine HIV/SARS-CoV-2 coinfected patients and found that low CD4^+^ T cell counts in the HIV-positive patients may adversely affect the COVID-19 outcomes [14]. Maggiolo compared 55 cases of SARS-CoV-2 infection with 69 asymptomatic people living with HIV (PLHIV) negative for SARS-CoV-2 RT-PCR and/or serology, and suggested that HIV-positive individuals are not protected from SARS-CoV-2 infection or have a lower risk of severe disease and those with low CD4^+^ T cell counts might have worse outcomes [15]. Studies also suggested that HIV-positive individuals should not be considered to be protected from SARS-CoV-2 infection or to have lower risk of severe disease [16, 17].

We described two cases of HIV and SARS-CoV-2 co-infection. Although there were no clinical clues of HIV/AIDS on admission of those patients, and the current treatment options for COVID-19 were limited, we treated those patients with a variety of treatments and it seemed not efficacious. So it raised another concern that whether the immune status of those patients were defected. Thus, the antigen/antibody combination tests of HIV were taken, which showed positive results, then we sent the blood samples of the patients to the local CDC (CDC of Caidian District) to recheck, they remained positive. According to the medical records, there was neither blood transfusion nor drug abuse through sharing of infected needles. However, they were likely MSM at high risk of HIV.

The process of confirmation test was held in Hubei provincial CDC, which showed a positive result, however, viral load test was delayed because of current local strict prevention measures. The patient was asked to receive the antiretroviral treatment and follow-up in a designated hospital, in accordance with local epidemic prevention law.

For the alternative diagnoses, other causes of pneumonia should be screened for and ruled out to ensure that data are accurate both to confirm the causative agent and to identify any co-infection that may exacerbate symptoms and severity of COVID-19. Cooper et al recommend that sputum and blood cultures should be taken early for detection of superimposed bacterial pneumonia and the presence of other causative agents [18]. There have been reported *Pneumocystis jirovecii* Pneumonia (PJP) cases among patients with HIV and COVID-19 co-infection [9, 19]. Patients with HIV show a higher incidence of bacterial pneumonia, which is inversely proportional to the CD4^+^ T cell count, when compared to the general population. Thus, superimposed bacterial pneumonia with COVID-19 is a significant consideration in PLHIV [20]. We admitted that these cases we reported could not rule out HIV-related opportunistic infections due to an urgent study and limit resource, we suspected but did not confirm PJP infection.

The current treatment options for COVID-19 are limited and may be not efficacious, clinical trials of Tocilizumab are ongoing [21, 22]. Fu et al suggested that Tocilizumab is an effective treatment in severe patients of COVID-19 to calm the inflammatory storm and reduce mortality [23]. Although there many other studies with Tocilizumab treatment that blocking IL-6 receptors showed inspiring clinical results [24–27], no benefits were received in our cases.

Our case report has some notable limitations. First, the results were based on only two patients, further large observational studies are needed to verify our results. Second, additional follow-up was not performed due to limit resource, however, as far as we know, fortunately, those two patients were discharged after being transferred to a designated hospital for critical care, they were administered with antiretroviral drugs (tenofovir, lamivudine and efaviren) in a HIV follow-up center and were both in stable condition. The efficacy of Tocilizumab for COVID-19 is still under investigation [28], so it is important to identify the immunological factors that are associated with SARS-CoV-2 infection in order to elucidate the pathogenesis of SARS-CoV-2 infection and prompt our understanding of this disease. The two cases we reported stressed the risk of misdiagnosis, especially during the pandemic of an infectious disease and the importance of extended testing even if in immune-compromised condition the immune state may be ignored.

## Supplementary information

**Additional file 1: Fig. S2.** Chest CT images of patient one on Feb 18. **Fig. S3.** Chest CT images of patient one on day 12. **Fig. S4.** Chest CT images of patient one on day 20. **Fig. S5.** Percentage change trend of lymphocytes and monocytes from patient one. **Fig. S6.** Change trend of serum albumin of patient one. **Fig. S7.** Chest CT images of patient one on day 25. **Fig. S8.** Change trend of pulmonary infection ratio of patient one calculated by chest CT artificial intelligence. **Fig. S9.** Chest CT images of patient two on day 10. **Fig. S10.** Chest CT images of patient two on day 19. **Fig. S11.** Change trend of pulmonary infection ratio of patient two calculated by chest CT artificial intelligence. **Fig. S12.** Change trends of C-reactive protein and serum albumin of patient two. **Fig. S13.** Percentage change trend of lymphocytes and monocytes from patient two.

## Data Availability

All the data regarding the findings are available within the manuscript. We have not shared the patient’s hospital records as they contain personal identification information.

## Abbreviations

SARS-CoV-2: Severe acute respiratory syndrome coronavirus 2
COVID-19: Coronavirus disease 2019
WHO: World health organization
HIV: Human immunodeficiency virus
RPR: Rapid plasma reagin test
PLHIV: People living with HIV
MSM: Men who have sex with men
PJP: *Pneumocystis jirovecii* pneumonia
