# Supplementary material for: New HIV diagnoses in patients with COVID-19: two case reports and a brief literature review
Source: BMC Infect Dis. 2020 Oct 19;20:771. doi: 10.1186/s12879-020-05480-y (PMC7570418; doi:10.1186/s12879-020-05480-y)
Supplement: Supplementary file 1 — Additional file 1: Fig. S2. Chest CT images of patient one on Feb 18. Fig. S3. Chest CT images of patient one on day 12. Fig. S4. Chest CT images of patient one on day 20. Fig. S5. Percentage change trend of lymphocytes and monocytes from patient one. Fig. S6. Change trend of serum albumin of patient one. Fig. S7. Chest CT images of patient one on day 25. Fig. S8. Change trend of pulmonary infection ratio of patient one calculated by chest CT artificial intelligence. Fig. S9. Chest CT images of patient two on day 10. Fig. S10. Chest CT images of patient two on day 19. Fig. S11. Change trend of pulmonary infection ratio of patient two calculated by chest CT artificial intelligence. Fig. S12. Change trends of C-reactive protein and serum albumin of patient two. Fig. S13. Percentage change trend of lymphocytes and monocytes from patient two. [file 12879_2020_5480_MOESM1_ESM.doc]

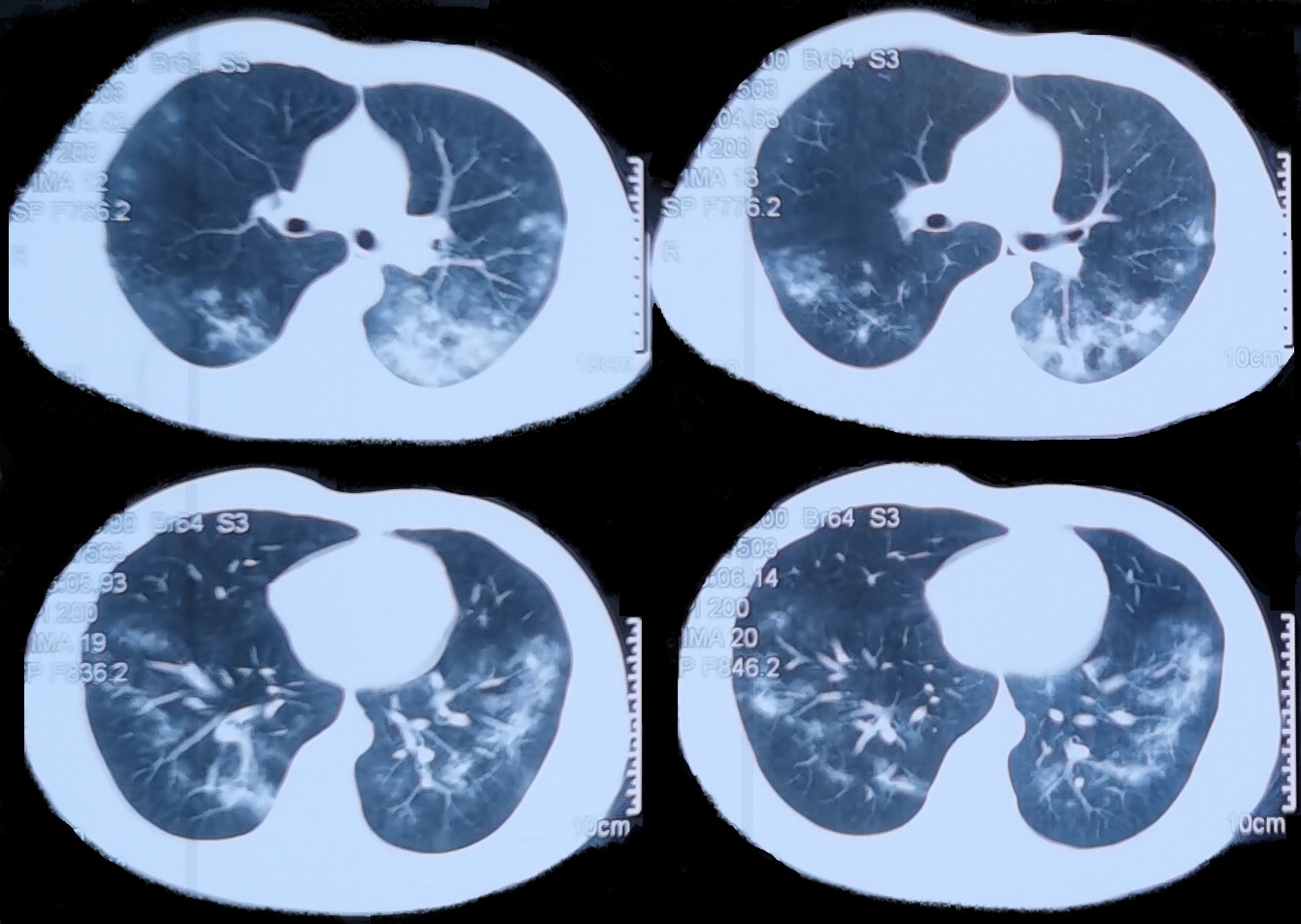


Figure 2. Chest CT images of patient one on Feb 18.


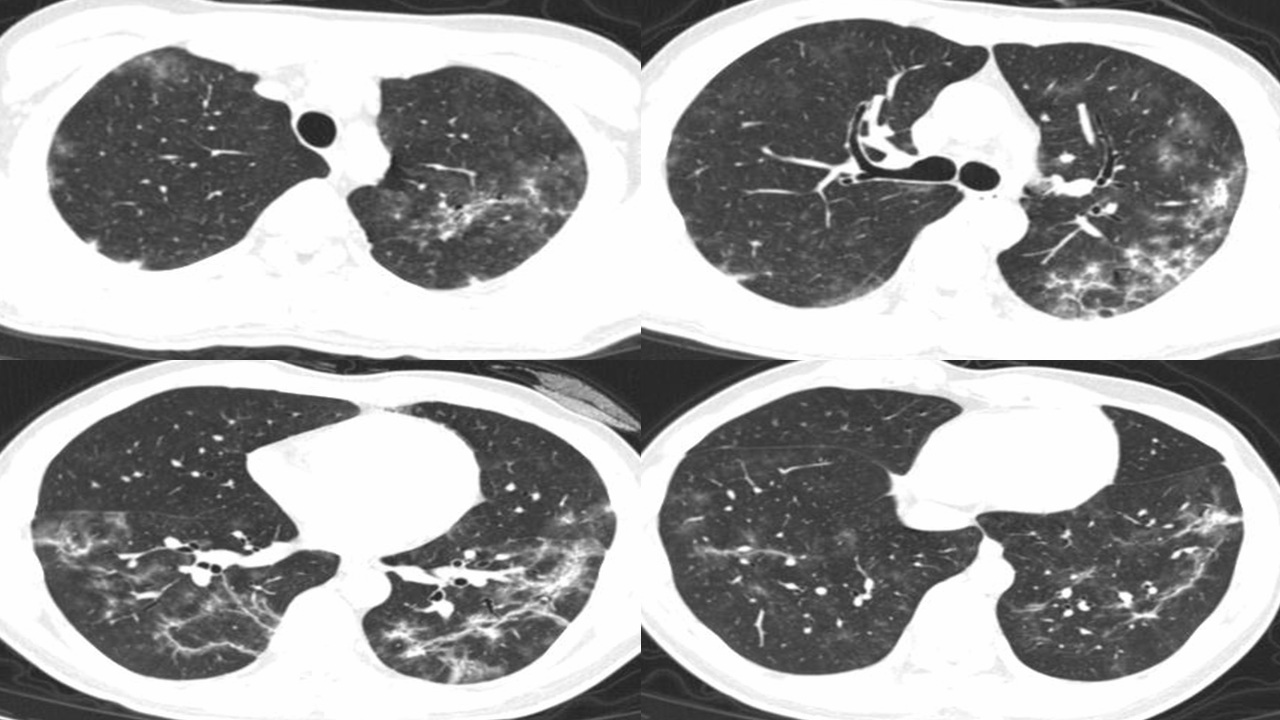


Figure 3. Chest CT images of patient one on day 12.


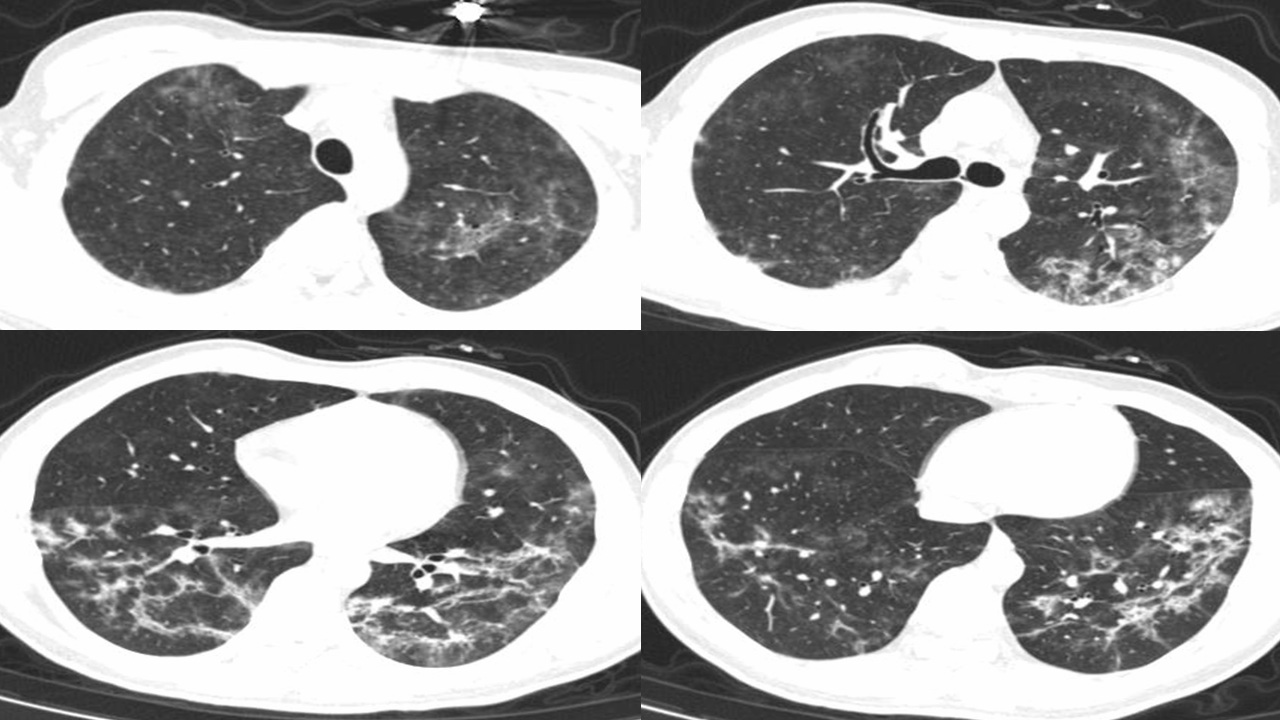


Figure 4. Chest CT images of patient one on day 20.


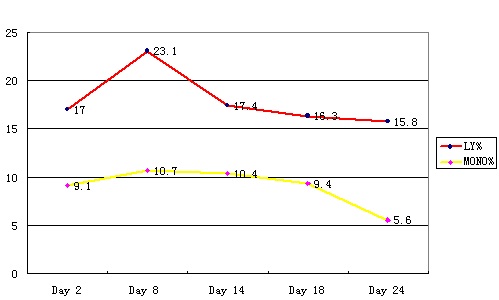


Figure 5. Percentage change trend of lymphocytes and monocytes from patient one.


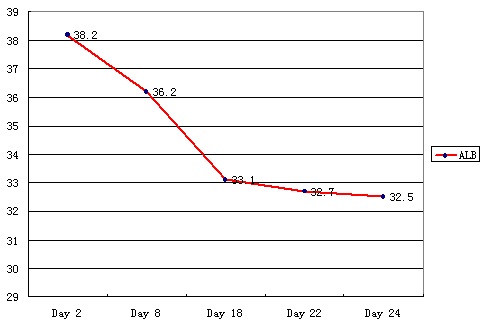


Figure 6. Change trend of serum albumin of patient one.


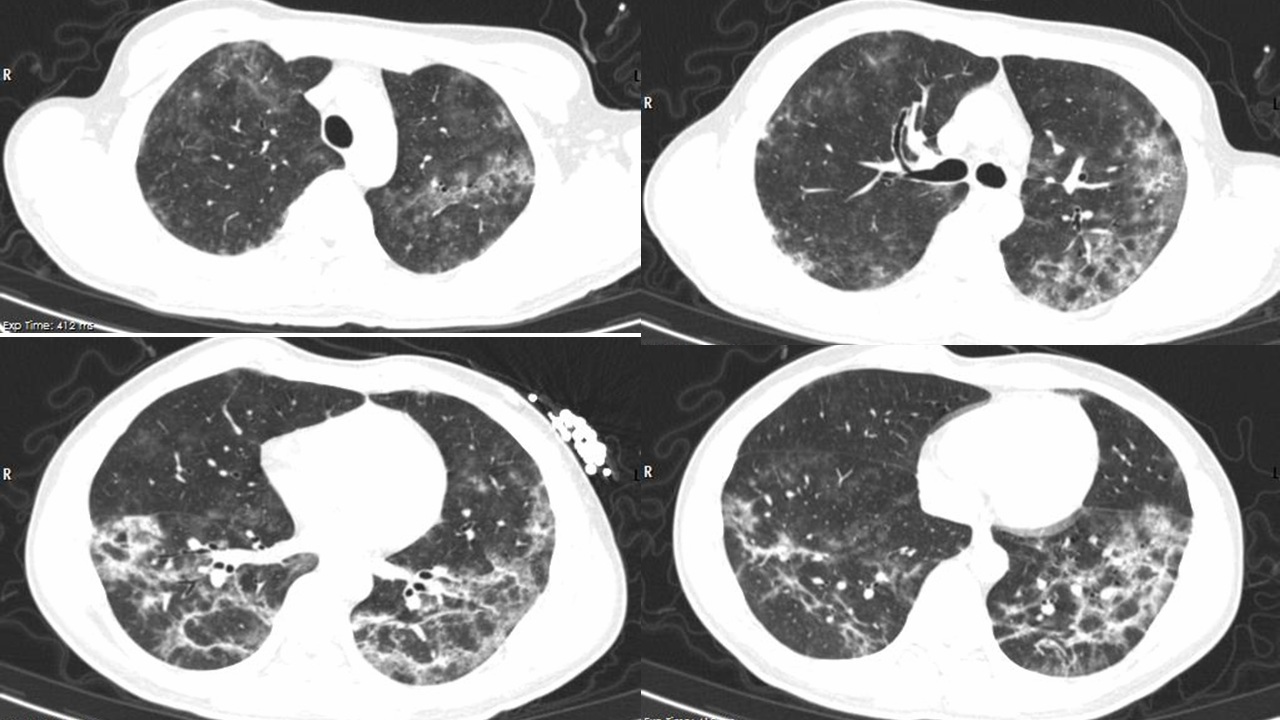


Figure 7. Chest CT images of patient one on day 25.


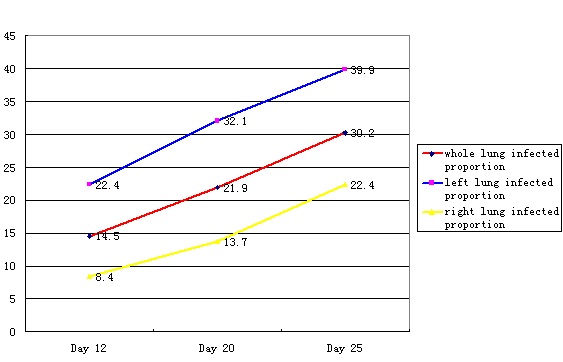


Figure 8. Change trend of pulmonary infection ratio of patient one calculated by chest CT artificial intelligence.


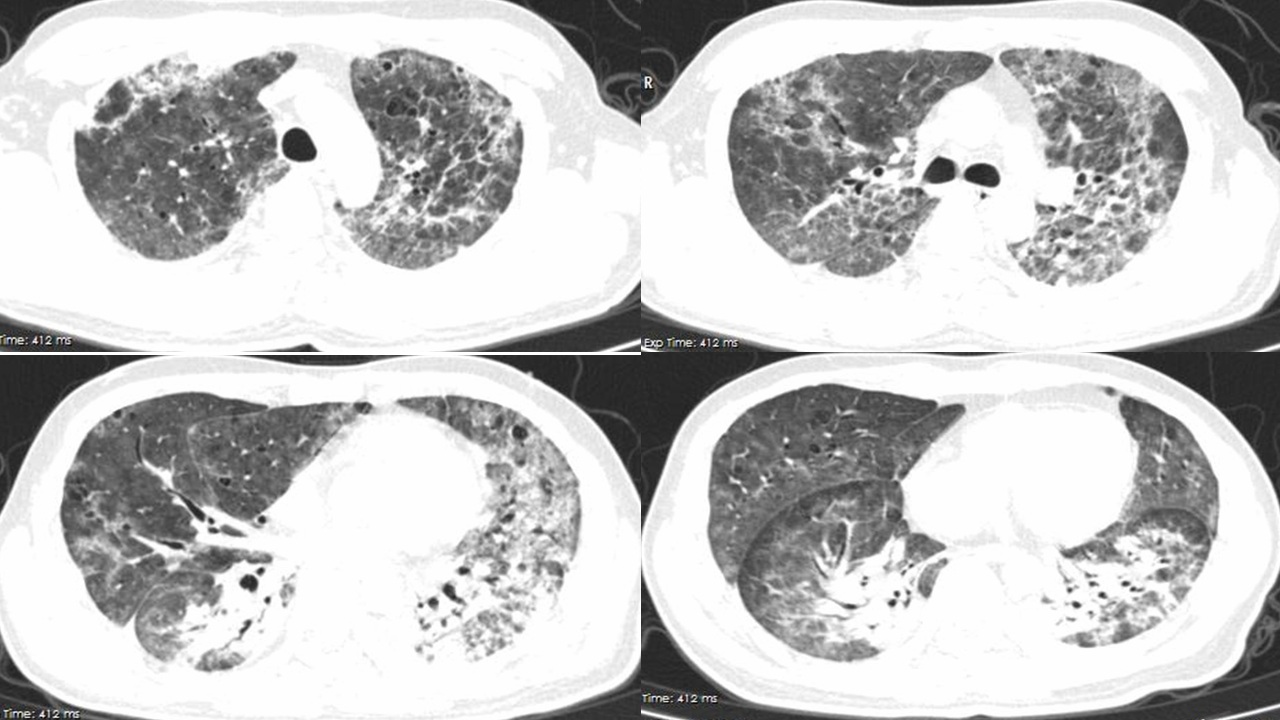


Figure 9. Chest CT images of patient two on day 10.


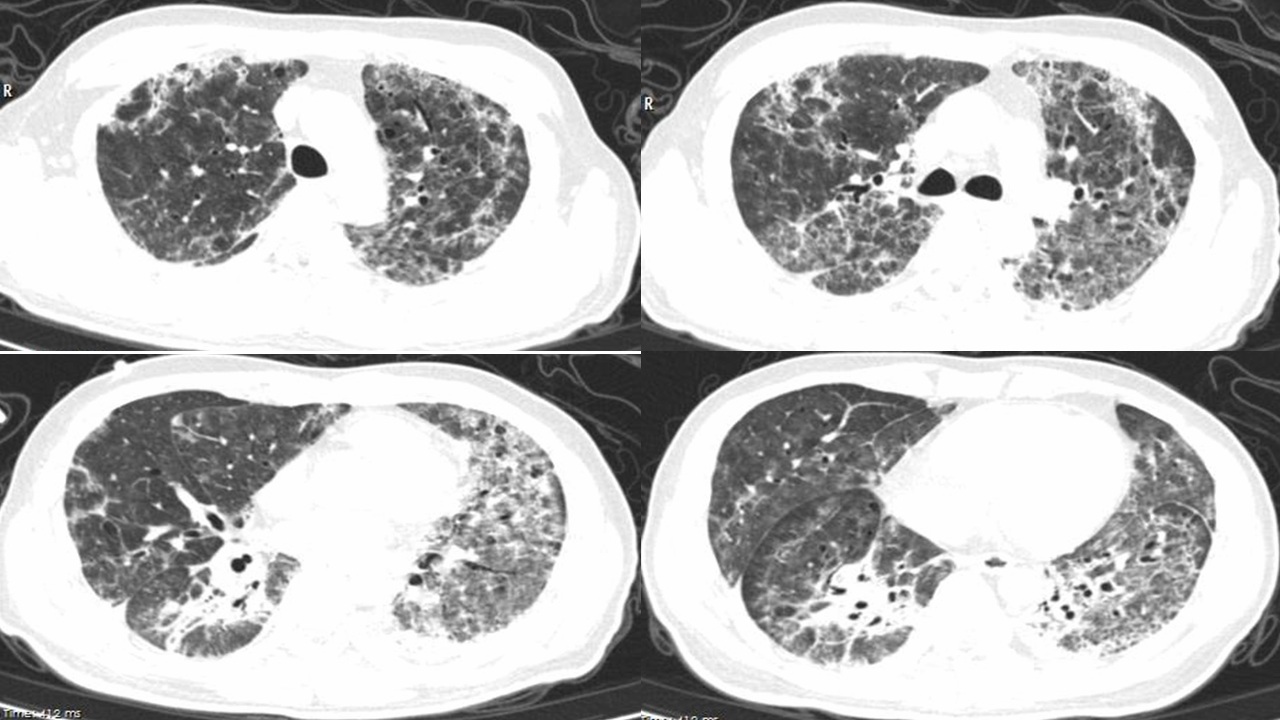


Figure 10. Chest CT images of patient two on day 19.


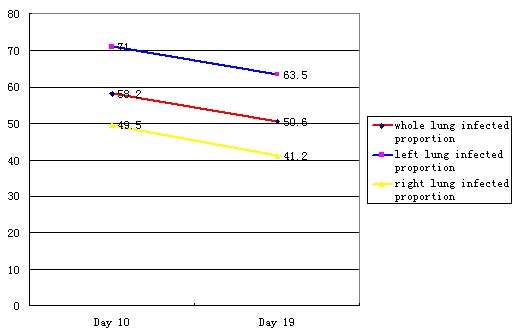


Figure 11. Change trend of pulmonary infection ratio of patient two calculated by chest CT artificial intelligence.


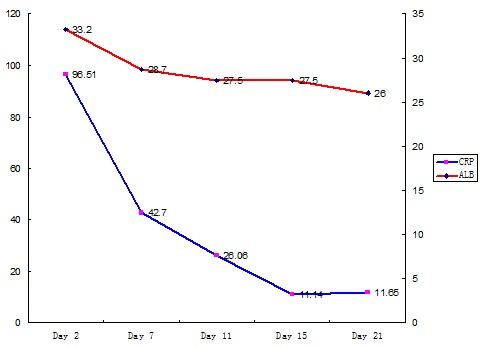


Figure 12. Change trends of C-reactive protein and serum albumin of patient two.


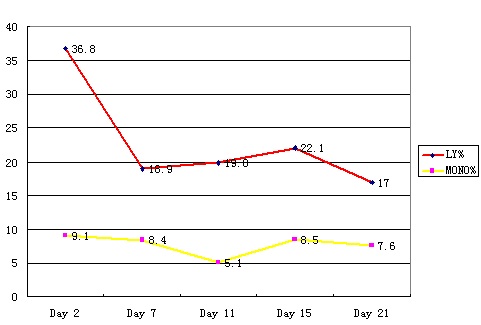


Figure 13. Percentage change trend of lymphocytes and monocytes from patient two.
